# Taxon-specific metagenomics of *Trichoderma* reveals a narrow community of opportunistic species that regulate each other’s development

**DOI:** 10.1099/mic.0.052555-0

**Published:** 2012-01

**Authors:** Martina A. Friedl, Irina S. Druzhinina

**Affiliations:** Research Area of Gene Technology and Applied Biochemistry, Institute of Chemical Engineering, Vienna University of Technology, Getreidemarkt 9-1665, A-1060 Vienna, Austria

## Abstract

In this paper, we report on the *in situ* diversity of the mycotrophic fungus *Trichoderma* (teleomorph *Hypocrea,* Ascomycota, Dikarya) revealed by a taxon-specific metagenomic approach. We designed a set of genus-specific internal transcribed spacer (ITS)1 and ITS2 rRNA primers and constructed a clone library containing 411 molecular operational taxonomic units (MOTUs). The overall species composition in the soil of the two distinct ecosystems in the Danube floodplain consisted of 15 known species and two potentially novel taxa. The latter taxa accounted for only 1.5 % of all MOTUs, suggesting that almost no hidden or uncultivable *Hypocrea/Trichoderma* species are present at least in these temperate forest soils. The species were unevenly distributed in vertical soil profiles although no universal factors controlling the distribution of all of them (chemical soil properties, vegetation type and affinity to rhizosphere) were revealed. *In vitro* experiments simulating infrageneric interactions between the pairs of species that were detected in the same soil horizon showed a broad spectrum of reactions from very strong competition over neutral coexistence to the pronounced synergism. Our data suggest that only a relatively small portion of *Hypocrea/Trichoderma* species is adapted to soil as a habitat and that the interaction between these species should be considered in a screening for *Hypocrea/Trichoderma* as an agent(s) of biological control of pests.

## Introduction

*Trichoderma* species are one of the most frequently isolated conidial fungi (teleomorph *Hypocrea*, Hypocreales, Ascomycota, Dikarya). They have been identified from a diverse range of natural and artificial substrata, which demonstrates their high opportunistic potential and adaptability to various ecological conditions (Druzhinina *et al.*, 2011). The exploitation of the latter properties of *Trichoderma* in biotechnology and agriculture mean that the genus has been well studied and it has been the focus of numerous *-*omic studies (see Lorito *et al.,* 2010 for a review), including the three complete genome sequences recently released for public access: *Hypocrea jecorina* (Martinez *et al.*, 2008), *H. virens* and *H. atroviridis* (Kubicek *et al.*, 2011). The comparative analysis of these genomes with each other and other Ascomycota revealed that the outstanding antagonistic ability of *Trichoderma* spp. against plant-pathogenic fungi (known as necrotrophic hyperparasitism or mycoparasitism) is the ancestral state for the genus (Kubicek *et al.*, 2011). Several studies have documented that *Trichoderma harzianum* *sensu* *lato*, *T. asperellum* and *T. asperelloides* are highly rhizosphere competent and are able to stimulate growth and immune defence of plants (reviewed by Harman *et al.*, 2004).

The current diversity of the holomorphic genus *Hypocrea*/*Trichoderma* is reflected in approximately 160 species (Samuels, 2005; Druzhinina *et al.*, 2006; Kubicek *et al.*, 2008, NCBI Taxonomy Browser, February 2011), the majority of which have been recognized on the basis of DNA sequence analysis and molecular phylogeny of pure cultures and/or herbaria specimens. A multigene phylogeny established in several studies (see Kubicek *et al.*, 2008, for a review and Jaklitsch, 2011, for updates) allowed the development of reliable tools for molecular species identification based on DNA barcoding, i.e. the analysis of DNA sequence polymorphism in the internal transcribed spacers 1 and 2 (ITS1 and 2) of the rRNA operon and other loci (Druzhinina *et al.*, 2005; Kopchinskiy *et al.*, 2005, http://www.isth.info).

The complete inventory of teleomorph-forming species in Central Europe indicated that there are 75 species (Jaklitsch, 2009, 2011). In addition to this, about 12 anamorphic species (for which no sexual stages are known) were reported in surveys of European soils and in taxonomic studies, as described by Wuczkowski *et al.* (2003), Kraus *et al.* (2004), Samuels *et al.* (2006b), Jaklitsch *et al.* (2006a, b), Komon-Zelazowska *et al.* (2007), Hagn *et al.* (2007), Druzhinina *et al.* (2008), Migheli *et al.* (2009), Zachow *et al.* (2009) and Meincke *et al.* (2010). Thus, the total number of *Hypocrea/Trichoderma* species identified so far in Europe may be about 100.

The major ecological niche for *Hypocrea/Trichoderma* may be deduced from the distribution of holomorphic species (when both teleomorphic and anamorphic stages are observed), which constitute the major genetic pool for the genus; they are associated with dead wood in different stages of its decay and with sporocarps of other fungi (Jaklitsch, 2009). *Trichoderma* species were initially believed to be among the dominant taxa inhabiting soil ecosystems (Klein & Eveleigh, 1998). However, this statement was not supported by the application of high-throughput sequencing methods to study the cultivation-independent fungal diversity in soil. These studies did not reveal that *Trichoderma* was abundant, listing it among the minor taxa (Buée *et al.*, 2009; Lim *et al.*, 2010).

Thus, molecular ecology and genomics of the genus indicate that the presence of *Hypocrea/Trichoderma* in soil, where it may either be a saprotroph or establish various associations with plants and animals (biotrophy), could be driven by the general mycotrophy, including various forms of mycoparasitism, combined with broad environmental opportunism (Rossman *et al.*, 1999; see Druzhinina *et al.*, 2011 for a review).

The aim of this research was to use a taxon-specific metagenomic approach to explore the diversity of *Hypocrea/Trichoderma* in a soil profile and to identify the factors which control the size/occurrence of its infrageneric communities.

## Methods

### 

#### Sampling sites.

Two sampling sites were chosen in the River Danube National Park ‘Nationalpark Donau-Auen’ (Austria). They were located 500 metres apart representing the essentially different biotopes (Table 1). The beech forest site (+48° 9′ 28 N, +16° 32′ 9 W, altitude 162 m) belongs to the hard wood riparian forest which is situated above the seasonal Danube overflow level (approx. 3 m above the water level). The aspen forest site (+45° 30′ 43 N, +73° 32′ 44 W, altitude 141 m) represents the softwood riparian forest regularly flooded during the seasonal Danube overflows. A detailed botanical description of both ecosystems is given in Table 1.

**Table 1.** Ecosystem description and soil properties

	Beech forest site				Aspen forest site		
Type of ecosystem	Hardwood riparian forest patch				Clearing of the soft wood riparian forest		
Flooding frequency	Rare to never				Regular		
**Botanical description**							
Trees	Tall, thick				Moderately tall, thin		
Dominant	*Ulmus laevis*, *Populus canescens*, *Fraxinus excelsior*				*P. alba*, *P. nigra*, *P. canescens*		
Rare	*P. nigra*, *P. alba*, *Salix rubens*				*S. rubens*		
Shrub and regeneration	Light				Dense		
Dominant	*Crataegus monogyna*				*Rubus idaeus*		
Rare	*Populus* sp., *Acer* sp., *Salix* sp.				*Quercus rubur*, *Populus* sp.		
Herbs	Lighted				Dense		
Dominant	*Aegopodium podagraria*				*Allium ursinum, Aegopodium podagraria*		
Rare	*Salvia glutinosa*, *Poaceae*, *Cyperaceae*				*Carex acuta*, *Urtica dioica*, *Epilobium* sp., *Salvia glutininosa*, *Silidago gigantea*		
Fallen trees	Abundant				None		
**Soil properties**							
Litter layer	Thick, well developed				Thin, undeveloped		
Soil type (FAO)	Cambic fluvisol				Calcaic fluvisol with a high level of sandy sediments		
Soil moisture content	Moderate				High		
Siltation	Moderate				High		
Vertical profile	Well differentiated				Well differentiated		
Horizon name	A0	A	B	C	A	BC	C
Thickness (cm; of 1 m section)	5	10	40–50	20+	10	20–30	50–60
Soil colour	Very dark brown	Dark greyish	Greyish brown	Very dark greyish brown	Light olive brown	Light olive brown	Light olive brown
Soil colour code (Munsell)	10YR 2/2	10YR 4/2	2.5Y 5/2	2.5Y3/2	2.5Y 5/3		
Relative root density	No roots	Very high	High	Moderate	High	Low	No roots
Chemical characteristics							
pH	7.77	7.90	8.10	8.24	7.60	8.15	8.23
C (%)	8.16	2.38	1.82	2.50	6.52	1.04	2.51
N (%)	0.37	0.15	0.06	0.04	0.43	0.07	0.04
C/N	22.05	15.87	30.33	62.50	15.16	14.86	62.75

#### Soil sampling and detection of soil properties.

In both ecosystems, pits about 100 cm deep were made and soil samples (~200 g) were taken from each soil horizon using a sterile knife. The soil samples were immediately separated from roots and large particles, and samples were air-dried and sieved through a metal mesh to collect fine particles <2 mm (‘fine earth fraction’). Thereafter, each soil sample was spread into a sterile tray and divided into four equally sized fragments, two of which were discarded while the remaining two were thoroughly mixed and again spread on the same tray for subsequent subsampling (Robertson *et al.*, 1999). Finally 50 g of each soil was stored at −20 °C for further molecular and chemical investigations.

Soil classification and the detection of soil horizons were done directly at the two sampling spots. The soil colour was defined using a standard colour scale for soil science (Munsell Soil Colour Charts, US Department of Agriculture). The horizons were visually distinguished based on their colour and were later differentiated based on their chemical properties. All chemical analyses were performed using the fine earth fraction. To measure pH, 1 g soil was suspended in 100 ml 1 M KCl and shaken for 1 h. Finally, pH was determined with a glass electrode. The total nitrogen content was determined according to the Kjeldahl method (see Batjes, 1996) on a Vapodest 30 (Gerhardt). The total organic carbon content was measured using the Liechtenfelder method (see Batjes, 1996), which oxidizes carbon with potassium dichromate and photometrically quantifies the generated Cr^3+^ (DIN 19684).

#### Development of genus-specific primers.

The genus-specific primers were developed based on the master alignment of ITS1 and 2 from 88 *Hypocrea* and *Trichoderma* reference strains (Druzhinina *et al.*, 2005) complemented by the new species described since that time (Jaklitsch *et al.*, 2005, 2006a, b; Overton *et al.*, 2006; Samuels *et al.*, 2006b; Komon-Zelazowska *et al.*, 2007; Jaklitsch *et al.*, 2008; Jaklitsch, 2009, 2011). All primers are reverse primers and are complementary to the forward primer ITS5 (5′-GGAAGTAAAAGTCGTAACAAGG-3′) (Table 2). The position of primers is shown on htttp://www.ISTH.info/methods/method.php?method_id=12. The verification of primer specificity was done with reference cultures described by Druzhinina *et al.* (2005). The general test of possible unspecific annealing was done by sequence similarity searches against NCBI GenBank (May 2006) adjusted for short query sequences.

**Table 2.** Genus- and species-specific PCR primers designed in this study

Primer	Locus	Application	Suggested pair	Specificity	Direction	Primer sequence
Trirev1	ITS2	ciPCR	ITS5	Genus, *Hypocrea* and *Trichoderma*	Reverse	5′-CATTTC(A/C)GAAAGTTGGGGTG-3′
Trirev2	ITS2	ciPCR	ITS5	Genus, *Hypocrea* and *Trichoderma*	Reverse	5′-CATTTC(A/C)GAAGTTTGGGGTG-3′
Trirev3	ITS2	ciPCR	ITS5	Genus, *Hypocrea* and *Trichoderma*	Reverse	5′-CATTTC(A/C)GAAAGTTTGGGTG-3′
Trirev4	ITS2	ciPCR	ITS5	Genus, *Hypocrea* and *Trichoderma*	Reverse	5′-CATTTC(A/C)GAAAGTTGGGTG-3′
Trirev5	ITS2	ciPCR	ITS5	Genus, *Hypocrea* and *Trichoderma*	Reverse	5′-CATTTC(A/C)GAAGTTGGGTG-3′
Trirev6	ITS2	ciPCR	ITS5	Genus, *Hypocrea* and *Trichoderma*	Reverse	5′-CATTTC(A/C)GAAGTTTGGTG-3′
citro1_64	*tef1*	ciPCR and qPCR	LLErev	Species, *H. schweinitzii*	Forward	5′-CGCTACTGCCTTCAGACCAC-3′
asp7_60	*tef1*	ciPCR and qPCR	LLErev	Species, *T. asperellum*	Forward	5′-GCTTTGCCAGTCTACCTACC-3′

#### Isolation of DNA and culture-independent PCR (ciPCR).

A representative subsample of the fine soil fraction (1 g) was thawed and dried overnight in a drying chamber at 90 °C. Afterwards, the individual soil samples were well homogenized and DNA was extracted using a FastDNA Spin kit for soil (MP Biomedicals). The DNA was then used as a template for ciPCR with *Hypocrea/Trichoderma*-specific rRNA primers as listed in Table 2. ciPCR products of all six primers were combined and purified using QiaQuick PCR purification kit (Qiagen).

ciPCRs were carried out in a total volume of 50 µl containing 2.5 mM MgCl_2_, 10 mM Tris/HCl pH 9.0, 50 mM KCl, 0.1 % (v/v) Triton X-100, 0.4 µM each primer, 0.2 mM each dNTP and 0.5 units *Taq* Polymerase (Promega). The amplification program consisted of 1 min initial denaturation (94 °C), 30 cycles of amplification (1 min 94 °C, 1 min 52 °C, 1 min 72 °C) and a final extension period of 7 min at 72 °C.

Amplification products were visualized on 1 % TAE agarose gels, and the ciPCR amplicons, at 600 kb, were excised, repurified using QiaQuick gel excision kit (Qiagen) and suspended in 20 µl water.

#### Construction of clone libraries and sequencing.

The ciPCR products were subcloned using the standard pGEMTeasy (Promega) procedure. At least 100 colonies were selected from each soil sample. Plasmids were extracted using a standard miniprep method. In order to determine whether the plasmid DNA was carrying the insert, the plasmid DNA was digested with 5 units of *Not*I and *Eco*RI (Fermentas). For positive samples, 0.5 µg plasmid DNA was finally used for automated sequencing (Eurofins) from both directions.

Representative alleles for all species from each horizon have been deposited in GenBank (accession numbers are given in the footnote on page 1). The alignment matrix is available upon request.

#### Species identification and diversity assessment.

All sequences were aligned in GeneDoc 2.6 (Nicholas & Nicholas, 1997) using the guidance for *Hypocrea/Trichoderma* ITS1 and 2 alignment provided by Druzhinina *et al.* (2005), available online at http://www.isth.info/tools/master.php. For species identification, the complete set of sequences was submitted to the DNA oligonucleotide barcode program *Trich*OKey (http://www.isth.info/tools/molkey/index.php; Druzhinina *et al.*, 2005). All sequences contained the set of five genus-specific hallmarks and were therefore attributed to *Hypocrea/Trichoderma*. Hence, tests for chimerical sequences were not required. All unusual ITS1 and 2 alleles have been further analysed by sequence similarity searches against NCBI GenBank, *Tricho*BLAST (Kopchinskiy *et al.*, 2005) and the sequence database of the collection of fungal strains of Vienna University of Technology that currently contains more than 4000 *Hypocrea*/*Trichoderma* strains with 5500 core nucleotide sequences including ITS1 and 2. Based on individual mismatches found in the otherwise conserved areas of ITS1 and 2 (for example, genus-specific hallmarks or 5.8S rRNA gene, see Druzhinina *et al.*, 2005), 20 % of the sequences have been found to contain single sequencing errors. Four sequences contained polymorphic sites in the diagnostic regions of both ITS1 and 2 and therefore have been diagnosed as potentially new alleles (*Trichoderma* sp. MOTU 1A 64 for section *Longibrachiatum* and *Trichoderma* sp. MOTU 2B 48 for section *Trichoderma*).

#### Development and verification of species-specific *tef1* primers for qPCR.

In order to design species-specific qPCR primers, representative sequences of the fourth large intron of the *tef1* gene coding the elongation factor 1 α for the whole genus *Hypocrea/Trichoderma* were retrieved from the multilocus database of phylogenetic markers (http://www.isth.info) and automatically aligned in clustal_x (Thompson *et al.*, 1997). The representative *tef1* sequence of a target species was submitted to NCBI sequence similarity search tool (blastn) and all homologous vouchered sequences (*n*≥20) attributed to the same species were retrieved and added to the initial alignment. Subsequently, species-specific diagnostic regions were manually selected for each target species so that they would contain the minimal level of infraspecific polymorphism. In parallel, the same alignments were used to design degenerate species-specific primers in hyden software (Linhart & Shamir, 2005). The annealing temperature and secondary structure of oligonucleotides designed based on both approaches have been estimated using Gene Runner (Gene Runner 3.0 software) and SMS PCR Primer Stats tools http://www.bioinformatics.org/sms2/pcr_primer_stats.html. The specificity of selected primers was first tested against the NCBI GenBank database (automatically optimized for short queries) and then verified by PCR (see Jaklitsch *et al.*, 2006a for conditions) with reference DNA from pure cultures of all genetically close members and several members of the neighbouring clades. The resulting potentially species-specific oligonucleotides were tested for annealing efficiency by applying serial dilutions of a target DNA extract. Selectivity of the designed primers was verified by subcloning the PCR product obtained from the soil DNA extract. Around 40 oligonucleotides have been synthesized to hit the diversity of the most frequent temperate *Hypocrea/Trichoderma* species but only two primers targeting *T. asperellum* and *H. schweinitzii* (Table 2) showed high specificity, selectivity and appropriate efficiency (≥80 %) for application to environmental samples.

#### Quantitative PCR assessment.

Quantitative PCR (qPCR) amplification was carried out by using the iQ 5 Real-Time PCR detection system (Bio-Rad) in a 25 µl reaction containing 12.5 µl iQ SYBR Green Supermix (Bio-Rad), each primer at 250 nM and sample, corresponding to an initial concentration of 0.5 µl total DNA. Amplification was carried out with the following PCR program: initial denaturation for 3 min at 95 °C, followed by 45 cycles of 95 °C for 15 s, 54.0 °C (*tef1*) for 20 s, and 72 °C for 20 s. Successful amplification was verified by determining the melting temperature and by agarose gel electrophoresis. For each species, a series of dilutions was performed to assess the efficiency of the PCR. The results of the real-time PCR were analysed with the iQ 5 optical system software (Bio-Rad). Using the PCR base line subtracted mode, the threshold cycle was calculated for all samples and the amplification efficiency for each primer was determined.

#### *In vitro* pair-wise interactions.

Strains were cultivated at room temperature and ambient illumination without direct sunlight in 100 ml Erlenmeyer flasks with 50 ml 3 % malt extract for 5 days without shaking. The mycelia developed mats on the surface of the liquid. Thereafter, the biomass was filtered out in aseptic conditions and the remaining cultivation solution was centrifuged at 25 155 ***g*** for 10 min to sediment the remaining spores. The supernatant was then filtered through 0.2 µm sterile cellulose acetate filter (Whatman) and used for the experiment. Aliquots of the final culture supernatant (50 µl) were then pipetted into 96-well microplates with a flat bottom profile and inoculated with 50 µl conidia suspension of the tested strain. Conidia of these were obtained by growing the strains on 3 % (w/v) malt extract agar for 7 days at room temperature and in ambient light. Conidia were harvested by rolling the sterile cotton swabs against the surface of the Petri plate and transferred to sterile 3 % malt extract. Spore concentration was standardized to 5×10^5^ ml^−1^ by turbidity measurements at 590 nm. Each microplate was inoculated with conidial suspension from one species. Microplates were then incubated at 25 °C in darkness for 8 days. Mycelial growth was measured every 24 h using a Biolog microplate reader by recording OD_750_. Conidiation was assessed visually after 192 h incubation. Complete experiments were repeated in eight linked replications (on one microplate) and in at least two independent repeats.

#### Statistical analysis.

Cultivation-independent diversity and species richness of *Hypocrea/Trichoderma* were assessed in EstimateS 8.2. Rarefaction curves were computed for each soil profile. The number of species was quantified for 100 random combinations of 1–n sequences and also by performing 100 bootstrap pseudoreplicates implemented in EstimateS (Colwell, 2005).

Statistical analyses of metagenomic data and *in vitro* infrageneric interactions were carried out using the Statistica 6.1 (StatSoft) software package using basic data exploration tools, correlation matrices, variance analyses and multifactorial techniques (factor analysis and cluster analysis). Discrete colour plots representing the results of pair-wise interactions between different *Hypocrea/Trichoderma* strains were constructed based on the results of a two-way joining cluster analysis implemented in Statistica 6.1 with consequent reordering of both variables and cases, in order to reflect phylogenetic groups inside the sample.

## Results

### The pedogenesis of both sites is strongly influenced by the river

Two sampling sites representing essentially different biotopes (Table 1) in a riparian forest were selected in the River Danube National Park, south-east of Vienna, Austria. We performed a 1 m deep vertical soil cut into both ecosystems in places that are equally distanced from surrounding big trees and shrubs and which therefore do not represent exclusive rhizospheres of any plant species.

The major difference between the two soil cuts was in the presence of the thick (min. 5 cm) soil litter layer (FAO horizon A0) in the beech site which was absent in the aspen forest. Instead, the latter site was covered by a dense herbal layer dominated by the blossoming *Alnus urcinum* with several codominant herbal species (see Table 1). Correspondingly, the beech forest soil profile was evenly penetrated by roots from different plants while in the aspen forest a high root density was only observed in the upper horizon.

The pedogenesis of both sites is strongly influenced by the river and results in formation of fluvisols (FAO classification: J; FAO, 1998) with a high level of calcaric soil material and sedimentation. The flooded soil in the aspen forest contains significantly more moisture than the beech forest (Table 1). Both sites were characterized by well-developed soil profiles and the presence of at least three horizons in FAO nomenclature. The soil of the beech forest was characterized by three clear horizons: A, B and C. At the aspen forest, the B layer contained little alteration products and was mixed with the parent material; therefore, the horizons at this sampling spot were classified as A, BC and C (Table 1). The chemical properties of the soil are detailed in Table 1.

### ciPCR revealed restricted diversity of *Hypocrea/Trichoderma* in temperate soil

We designed a set of genus-specific ciPCR reverse primers Trirev (1–6) (5′-CATTTCMG[A_2_/A_3_]G[T_2_/T_3_][G_2_/G_3_/G_4_]TG-3′, melting temperature 61 °C; Table 2) at the 3′-end of the ITS2 of the rRNA gene cluster (Table 2). The position of the primers is illustrated on the ISTH website: http://www.isth.info/methods/method.php?method_id=12. When applied together with the ITS5 forward primer (White *et al.*, 1990), these primers amplified a 510–540 bp fragment covering the complete diagnostic area of ITS1 and 2 including all five genus-specific hallmarks (Druzhinina *et al.*, 2005).

Application of the ITS5/Trirev primer pair in a ciPCR with DNA extracts from soil samples showed different results for individual primers and also differing efficiency of the Trirev primers in samples from different horizons. The highest affinity of Trirev primers was detected for horizon A followed by the two other soil horizons of the beech forest site (Supplementary Fig. S1, available with the online version of this paper). PCR efficiency in the soil of the aspen forest was lower. The pooled ciPCR products of ITS5/Trirev obtained for each soil horizon were used for subsequent clone libraries.

**Fig. 1.**
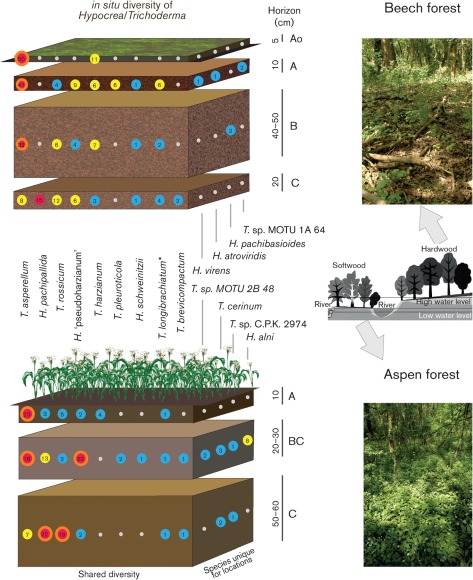
*In situ* diversity of *Hypocrea/Trichoderma* in the vertical profiles of two soil cuts in a hardwood beech and a softwood aspen forest. Numbers in coloured circles indicate the number of MOTUs of each species recovered; red, yellow and blue circles correspond to dominant, subdominant and rare species, respectively. The schematic profile of the flood plain ecosystem of the River Danube (middle) and images of the sampling sites (top and bottom) are shown to the right. *, T. longibrachiatum–*H. orientalis* species pair.

In total, 411 ITS1 and two rRNA molecular taxonomic units (MOTUs) were recovered (Fig. 1). All sequences had diagnostic genus-specific hallmarks (Druzhinina *et al.*, 2005), suggesting that the Trirev primers were highly selective. All MOTUs were identified as being one of 15 known species or two putatively novel taxa (Table 3). The rarefaction curve suggested that the species richness was close to saturation, as an additional 100 MOTUs revealed only one or two additional species (Supplementary Fig. S2, available with the online version of this paper).

**Table 3.** *In situ* diversity of *Hypocrea/Trichoderma* in vertical soil profile and its correlation with soil properties The total size of the clone libraries was 243 and 168 for the beech and aspen forests, respectively. The diversity index for each soil horizon category was calculated as the ratio between the number of taxon-attributed MOTUs (as given in Fig. 1) to the total number of MOTUs per soil horizon. Bold type indicates significant product–moment correlation coefficients, *P*<0.05; italic type indicates significant coefficients for repressed species. TF, Total frequency; LF, local frequency.

Species	TF (%)	Beech forest					Aspen forest				Correlation coefficients						
		LF (%)	Soil horizon				LF (%)	Soil horizon			Soil horizon	pH	C (%)	N (%)	C/N	Root density	Depth (cm)
			A0 (61)*	A (77)	B (54)	C (51)		A (38)	BC (75)	C (55)							
*T. asperellum*	43.6	53.9	0.206	0.169	0.132	0.033	28.6	0.137	0.107	0.042	**−1.0**	**−0.8**	–	–	**−0.8**	–	**0.9**
*H. pachypallida*	12.9	6.2	–	–	–	0.062	22.6	0.018	0.077	0.131	**0.8**	–	–	–	–	–	–
*T. rossicum*	11.7	9.1	–	0.016	0.025	0.049	15.5	0.030	0.012	0.113	–	–	–	–	**0.8**	–	–
*H.* ‘pseudoharzianum’	11.2	7.8	–	0.037	0.016	0.025	16.1	0.012	0.137	0.012	–	–	–	–	–	–	–
*T. harzianum* *sensu stricto*	7.5	11	0.045	0.025	0.029	0.012	2.4	0.024	–	–	–	–	–	–	–	–	–
*T. longibrachiatum*–*H. orientalis*	3.6	5	–	0.025	0.008	0.016	1.8	0.006	0.006	0.006	–	–	–	–	–	**0.9**	–
*H. alni*	1.9	–	–	–	–	–	4.8	–	0.048	–	–	–	–	–	–	–	–
*T. pleuroticola*	1.9	2.5	–	0.025	–	–	1.2	–	0.012	–	–	–	–	–	–	–	–
*T. cerinum*	1.2	–	–	–	–	–	3.0	–	0.018	0.012	–	–	–	–	–	–	–
*T. brevicompactum*	1.0	0.8	–	–	–	0.008	1.2	–	0.006	0.006	*0.9*	*0.8*	–	–	–	–	*−0.8*
*H. schweinitzii*	1.0	1	–	0.004	0.004	0.004	0.6	–	0.006	–	–	–	*−0.8*	–	–	–	–
*Trichoderma* sp. C.P.K. 2974	0.5	–	–	–	–	–	1.2	–	0.006	0.006	–	–	–	–	–	–	–
*Trichoderma* sp. MOTU 2B 48 section *Trichoderma*	0.5	–	–	–	–	–	1.2	–	0.012	–	–	–	–	–	–	–	–
*H. pachybasioides*	0.5	0.8	–	–	0.008	–	–	–	–	–	–	–	–	–	–	–	–
*Trichoderma* sp. MOTU 1A 64 section *Longibrachiatum*	0.5	0.8	–	0.008	–	–	–	–	–	–	–	–	–	–	–	–	–
*H. atroviridis*	0.2	0.4	–	0.004	–	–	–	–	–	–	–	–	–	–	–	–	–
*H. virens*	0.2	0.4	–	0.004	–	–	–	–	–	–	–	–	–	–	–	–	–
Total	100	100					100										
Diversity index	1.0	0.8	0.15	0.77	0.34	0.62	0.8	0.46	0.92	0.62	0.5	0.5	−0.7	−0.5	0.0	0.4	−0.3

* The size of each horizon-specific clone library is given in parentheses.

MOTU frequencies were used to calculate the diversity index as an indirect quantitative measure of community composition. The dominant species in both sites (54 and 28.6 % for beech and aspen forests, respectively) was *T. asperellum* *sensu* *stricto* (Samuels *et al.*, 2010).

The beech forest soil was co-dominated by *T. harzianum* *sensu* *stricto* (Druzhinina *et al.*, 2010b), *T. rossicum*, *H*. ‘pseudoharzianum’ (Druzhinina *et al.*, 2010b, widely known as *T. harzianum* complex) and *H*. *pachypallida* (Jaklitsch, 2011) which contributed 11, 9, 8 and 6 %, respectively, of the total diversity at this location. Sequences of the *T. longibrachiatum*–*H. orientalis* species pair (Druzhinina *et al.*, 2008) added 5 % and could be considered as a subdominant taxon (Fig. 1). *T. pleuroticola* (2.5 %), *H. schweinitzii* (1.2 %), *T. brevicompactum* (0.8 %), *H. pachybasioides* (0.8 %) and taxonomic units of a putative novel species from section *Longibrachiatum Trichoderma* cf. sp. nov. MOTU 1A 64 (0.8 %) were rare. MOTUs of *H. atroviridis* and *H. virens* were recovered with a frequency <0.5 %.

A qualitatively similar but quantitatively different species composition was observed in the aspen forest (Fig. 1): the dominant *T. asperellum* was followed by co-dominant *H*. *pachypallida* (23 %), *H*. ‘pseudoharzianum’ (16 %) and *T. rossicum* (15.5 %). The subdominant species *H. alni* (5 %) and *T. cerinum* (3 %) were present in significantly lower amounts. *T. harzianum* *sensu* *stricto* (2.4 %), *T. longibrachiatum*–*H. orientalis* species pair (1.8 %), *T. pleuroticola* (1.2 %), *T. brevicompactum* (1.2 %), *Trichoderma* sp. C.P.K. 2974 (I. S. Druzhinina, unpublished data) (1.2 %) and a putative novel species from section *Trichoderma* sp. nov. MOTU 2B 48 were rare; the ITS sequence of *H. schweinitzii* was recovered only once.

Thus, among 17 recovered taxa, we detected nine species present at both sites and four species unique to each ecosystem. In total, soils in both forest sites supported the community of at least 13 co-existing *Hypocrea/Trichoderma* species, although these communities were unequal in two locations.

### The highest *Hypocrea/Trichoderma* diversity was detected in the moist horizon of aspen forest soil that has a low root density and poor carbon content

The analysis of *Hypocrea/Trichoderma* species distribution in the vertical soil profile showed that *T. asperellum* was most abundant in the soil litter layer of the beech forest (82 %) and also dominated in A and B horizons (53 and 59 %, respectively) while in horizon C it was only the third most abundant taxon (16 %). A similar distribution pattern was observed in the aspen forest soil (Table 3). The correlation analysis showed a significant negative correlation of *T. asperellum* abundance with pH values (Table 3) that decreases with depth, indicating that this species is associated with either the soil litter layer or the upper organic horizon A.

The distribution of the next most frequent species *H. pachypallida* in the vertical soil profile is inversely proportional to that of *T*. *asperellum* (Fig. 1, Table 3); in the beech forest soil it dominated the deepest C horizon (30 %) and was not detected in others, while in the aspen forest it was found in all horizons but with increasing frequency from top to bottom. The significant correlation of its occurrence with the horizon (Table 3) but not with other factors (carbon content, pH and depth) suggests the presence of yet another parameter which controls the distribution of this species and which was not monitored in this study.

*T. rossicum*, one of the co-dominant species in both locations, is also more frequent in the deeper soil horizons showing a significant positive correlation with the C/N ratio (Table 3). MOTUs of the *T. longibrachiatum*–*H. orientalis* species pair were the most frequent in the A horizon of the beech soil profile which has the highest root density (*r* = 0.8, *P*<0.05). No correlations were detected for *H*. ‘pseudoharzianum’ and *T. harzianum* *sensu* *stricto*.

The highest diversity of *Hypocrea/Trichoderma* (12 coexisting species) was found in the moist BC horizon of the aspen forest soil which has a low root density and the lowest carbon content. The next most diverse infrageneric community (10 species) was in the topsoil horizon A in the beech forest, which has the highest root density. As these communities share six species but have no similar frequencies, this indicates that no significant correlation between species richness and soil properties were detected.

We applied several statistical techniques to reveal possible correlations between the distribution of MOTUs from different species. The product–moment correlation analysis confirmed the significant (*P*<0.05) negative correlation between *T. asperellum* on one hand and *H. pachypallida* and *T. rossicum* on the other (Supplementary Table S1, available with the online version of this paper). No other significant correlation between dominant (co- and sub-) species was revealed, suggesting that these species have different responses to the microecological conditions.

### qPCR confirms the vertical distribution of *T. asperellum*

**Fig. 2.**
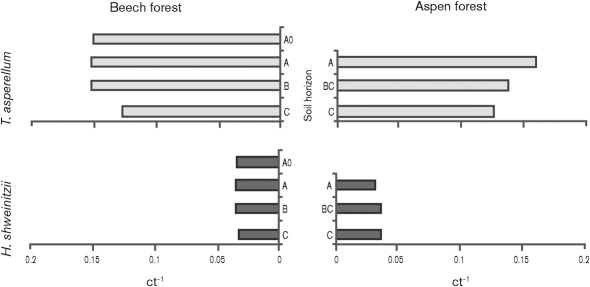
Results of the qPCR analysis based on species-specific *tef1* primers developed for *T. asperellum* and *H. schweinitzii*. As the efficiency of primers binding the environmental (soil) DNA was slightly different between these species, only a semiquantitative comparison is possible. The number of threshold cycles/1 (ct^−1^) corresponds to the abundance of the target DNA in the sample.

In order to have a second means of testing species distribution in the soil profile, we designed species-specific primers for qPCR based on the polymorphic fragment of the *tef1* gene. Although 40 primers were tested (see Methods for details), only those for *T. asperellum* and *H. schweinitzii* were shown to be highly selective and efficient when applied to soil DNA extracts (Table 2, Fig. 2). Using these, we detected a higher abundance of *T. asperellum* and a relatively small amount of *H. schweinitzii* DNA in all seven samples, which is in accordance with the metagenomic data. It was also possible to detect a reduction in *T. asperellum* biomass with depth while the distribution of *H. schweinitzii* remained constant.

### *Hypocrea/Trichoderma* species respond in a unique way to the presence of their tribal relatives

**Fig. 3.**
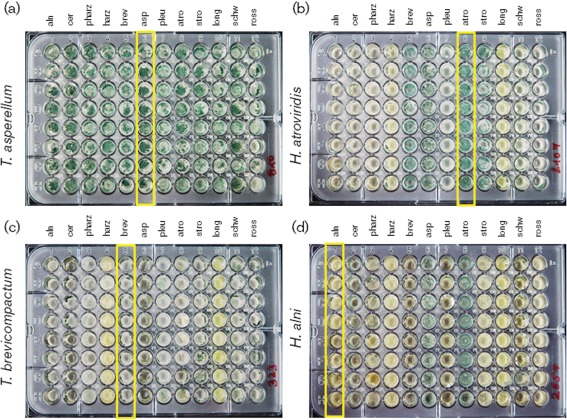
Selected examples of four 96-well microplates inoculated with spores of *T. asperellum* (a), *H. atroviridis* (b), *T. brevicompactum* (c) and *H. alni* (d) after 192 h incubation in darkness at 25 °C. Vertical columns correspond to repeated applications of culture extracts of *H. alni* (aln), *T. cerinum* (cer), *H*. ‘pseudoharzianum’ (pharz), *T. harzianum* (harz), *T. brevicompactum* (brev), *T. asperellum* (asp), *T. pleuroticola* (pleu), *H. atroviridis* (atro), *H. stromatica* (stro), *T. longibrachiatum* (long), *H. schweinitzii* (schw) and *T. rossicum* (ross), as indicated in Table 4. Control cases, when cultures were grown on their own culture extracts, are boxed.

**Fig. 4.**
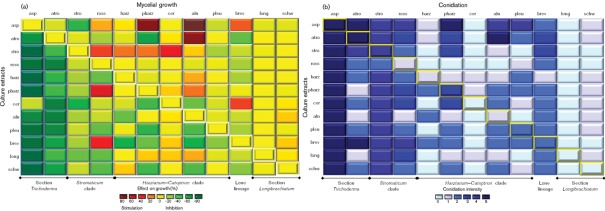
Discrete colour plots showing the results of a pair-wise modelling of *Hypocrea/Trichoderma* communities when selected strains were grown in the presence of a culture extract of any other strain in the experiment. Diagonal values correspond to controls when cultures were grown on their own culture extracts. (a) Effect on growth shown as % growth inhibition (greens) or growth stimulation (reds). Values are normalized to the control. (b) Effect on conidiation expressed in a nominative scale developed by Friedl *et al.* (2008). Complete absence of conidiophores and conidia (0), formation of immature pustules without mature conidia (1) and four levels of conidiation intensity (2–5) from weak diffuse sporulation (2) up to the development of a thick conidial mat covering the whole well (5). Values are not normalized to the control. Rows/columns are labelled as described in the legend for Fig. 3.

We show that several common *Hypocrea/Trichoderma* species may co-exist in one horizon of a floodplain soil. As many of these species are known for their mycoparasitic activity (for example *T. asperellum*, *H. atroviridis*, *T. rossicum*, *T. harzianum sensu stricto*, *H.* ‘pseudoharzianum’ or *T. pleuroticola*) they may also compete with their tribal relatives or, alternatively, they may behave synergistically. In order to determine this, we randomly selected 11 strains available in the collection of industrial micro-organisms from Vienna University of Technology (TUCIM) that were of temperate origin and represented species that were detected as one community in this study. We also included the *ex-*type strain of *T. stromaticum* (teleomorph *H. stromatica*) as a member of a tropical ecosystem (isolated associated with *Moniliophthora perniciosa*, a causative agent of ‘witches' broom’ of *Theobroma cacao*; Samuels *et al.*, 2000) because this species is closely related to *T. rossicum* (Table 4). Conidia of each of these species were grown in the presence of culture extracts of all other species in pair-wise combinations (Fig. 3). The results show that the strains express strongly differentiated responses to the presence of culture extracts from their close tribal relatives. Strains of *T. asperellum* and *H. atoviridis* (both belonging to section *Trichoderma*), which have the fastest growth rate (Supplementary Fig. S3, available with the online version of this paper), showed a strong inhibition (up to 90 %) of their mycelial growth rate in the presence of culture extracts of any other species (Fig. 4a). Two other closely related strains (*T. longibrachiatum* and *H. schweinitzii*, section *Longbrachiatum*) showed nearly no response to the presence of any other culture extract. *H. stromatica* and *T. rossicum* (both members of the phylogenetic *Stromaticum* clade) differed in their response to other fungi: the first species was either insensitive or slightly inhibited (~20 %) while the latter was significantly stimulated by the presence of culture extracts of *T. asperellum* (20 %), *T. stromaticum*, *H*. ‘pseudoharzianum’ and *T. brevicompactum* (50 % each). The most versatile response was detected for strains representing the *Harzianum*–*Catoptron* clade: in general these species are either insensitive to culture filtrates from other fungi (*T. pleuroticola*) or their growth is strongly stimulated. Thus, *H. alni* formed 180 % of its biomass in the presence of culture extract of *T. asperellum* and 85 % in the presence of *H. atroviridis* (Fig. 4a). Only a few cases of growth inhibition were found for this group: *H*. ‘pseudoharzianum’ grew poorly in the presence of culture extract of *H. atroviridis*, indicating competitive relations between these strains. *T. brevicompactum* was slightly inhibited by all other cultures except *T. asperellum* and *T. cerinum*, which caused 25 and 28 % acceleration, respectively.

**Table 4.** Strains of *Hypocrea*/*Trichoderma* selected for *in vitro* modelling of tribal interactions

Species	Strain	Origin	ITS1 and 2 GenBank accession no.
*T. asperellum*	C.P.K. 820, CBS 433.97 *ex-*type	USA	GQ495671
*H. atroviridis*	C.P.K. 2107, SzMC 3107	Hungary	GU111561
*H. stromatica*	C.P.K. 386, CBS101865 *ex-*type	Brazil	FJ442675
*T. rossicum*	C.P.K. 889, MA 2480	Austria	AJ507082
*T. harzianum* *sensu stricto*	C.P.K. 1426	Russia	GU111562
*H. ‘*pseudoharzianum’	C.P.K. 2973	Austria	GU111563
*T. cerinum*	C.P.K. 3034	Austria	GU111564
*H. alni*	C.P.K. 2657, UNISS 10-16	Italy	EF488139
*T. pleuroticola*	C.P.K. 2104, CBS 121145	Hungary	EF392794
*T. brevicompactum*	C.P.K. 323, MA 3295	USA	AY324173/AY324183
*T. longibrachiatum*	C.P.K. 2062, CECT 2412	UK	EU401572
*H. schweinitzii*	C.P.K. 3619	Russia	GU111565

The presence of culture extracts of tribal relatives also significantly influenced the conidiation (Figs 3 and 4b), whereas the conidiation of *T. asperellum*, whose growth was inhibited by presence of all other culture filtrates, was completely insensitive to the extracts of all other fungi (Fig. 4b). *H. atroviridis*, intensively conidiating in the control experiment, almost stopped producing conidia in the presence of *T. longibrachiatum*, *H. alni* and *H.* ‘pseudoharzianum’. In contrast, the poorly sporulating *T. rossicum* produced abundant conidia in the presence of nearly all other culture extracts except for that of *H. alni*. Many strains showed a versatile response ranging from suppression to stimulation of conidiation (*H.* ‘pseudoharzianum’, *T. pleuroticola*, *T. brevicompactum*; Fig. 4b).

Statistical analysis revealed only a few cases of significant correlation (*P*<0.05) between the effects of tribal relatives on growth and conidiation respectively (Supplementary Table S2, available with the online version of this paper). The majority of the strains respond in a unique way to the presence of the culture extracts of other *Hypocrea/Trichoderma*. Similarly, the effects on growth and conidiation were looked at, positive correlations were only seen for *H. alni* (*r* = 0.59) and *H. atroviridis* (*r* = 0.62) and there was a negative correlation for *T. harzianum* *sensu stricto*, indicating that these processes are differently controlled in every *Hypocrea/Trichoderma* community.

## Discussion

To study the generic community of *Hypocrea/Trichoderma* in soil we selected a riparian forest in the River Danube National Park. This ecosystem is unique as it still resembles the original European river floodplain landscape free from anthropogenic loadings (Wuczkowski *et al.*, 2003). The forest is characterized by high biodiversity, including several otherwise rare plant species (Tockner *et al.*, 1998).

### Temperate soil supports a limited diversity of highly opportunistic *Hypocrea/Trichoderma* spp.

Although the biodiversity of higher fungi is considered to be largely unknown (Hawksworth, 1991), and studies using cultivation-independent methods should thus result in the identification of a high percentage of unknown taxa, this is not the case with *Hypocrea/Trichoderma*. Our data suggest that, at least in soils of temperate climate, there is almost no hidden diversity of *Hypocrea/Trichoderma*: among 411 MOTUs, 407 were safely attributed to 15 existing species or to putative novel taxa that have previously been sampled. The diversity of *Hypocrea/Trichoderma* in Europe consists of at least 75 holomorphic species (Jaklitsch, 2009) and 10–20 anamorphic species (see Introduction), approaching 100 taxa. We found only a minor portion of the potentially expected diversity (roughly 15 %) which is in agreement with the previous hypothesis that soil itself is not the primary ecological niche for the genus (Druzhinina *et al.*, 2011). A similar outcome was also obtained by using the pioneering metagenomic studies of *Hypocrea/Trichoderma*. Hagn *et al.* (2007) used the ITS1 fragment and found only about 12 species in arable soil. Meincke *et al.* (2010) used partial ITS1 and 2 sequences and detected about 20 *Hypocrea/Trichoderma* taxa in the rhizosphere of *Solanum tuberosum*, although no positive species identifications were made. This view is also in agreement with studies that applied high-throughput sequencing to reveal the actual *in situ* diversity of higher fungi in soil; in these studies, *Hypocrea/Trichoderma* MOTUs were only found in minor proportions compared with other dominant groups of *Ascomycota* (Buée *et al.*, 2009; Lim *et al.*, 2010).

The perception of *Hypocrea/Trichoderma* as a common soil fungus is based on the abundant isolates from soil samples worldwide. However, the qualitative analysis of the diversity revealed in such samples shows dominance by the same 15–20 highly opportunistic species such as *T. asperellum*, *T. asperelloides*, *T*. cf. *harzianum*, *T. hamatum*, *T. atroviride*, *T. virens*, *T. longibrachiatum*, *T. gamsii*, *T. citrinoviride*, *T. koningiopsis*, *T. spirale*, *T. koningii* complex etc. (see http://www.isth.info/materials/topic.php?material_id=42 for details), which probably obtained the ability for saprotrophic growth in soil due to their general outstanding opportunistic potential, as suggested based on the genomes of *H. atroviridis* and *H. virens* (Druzhinina *et al.*, 2011; Kubicek *et al.*, 2011). Moreover, the antifungal activity of *Hypocrea/Trichoderma* spp. favours their detection as they are able to suppress other fungi. The view of soil as the main determinant of the *Hypocrea/Trichoderma* ecological niche is not supported.

The qualitative composition of the *Hypocrea/Trichoderma* community reveals that soil is inhabited by highly opportunistic species with cosmopolitan distributions as all taxa, except the three MOTUs of putative novel species, are common and have been identified from multiple isolates from numerous substrata (including soil) from temperate ecosystems worldwide.

### The occurrence of *Hypocrea/Trichoderma* species in different soil profiles is not determined by root density

The PCR efficiency with *Hypocrea/Trichoderma*-specific primers was essentially higher in samples from the moist beech forest (all horizons except the litter layer A0) compared with those from the aspen site (Supplementary Fig. S1), while there was no visual difference between both sites when general fungal primers were used (data not shown). This finding indirectly suggests that the beech forest contains more *Hypocrea/Trichoderma* biomass than the aspen soil.

Not all species known to be abundant in this region have been detected in soil profiles. For instance, the most frequent teleomorphic *Hypocrea/Trichoderma* species in Central Europe, *H. minutispora* (Jaklitsch, 2009), was not identified in our study at all. Despite this, *H. minutispora* was found in abundance in air samples from nearly the same region (within a few km^2^) using the same PCR methodology as here (M. A. Friedl, I. S. Druzhinina, unpublished data), thus supporting the assumption that there was no methodical bias in our experimental procedures. Similarly, many other very common local *Hypocrea* species (e.g. *H. viridescens*, *H. rufa*, *H. pulvinata*, *H. strictipilosa*; Jaklitsch *et al.*, 2006a, b; Overton *et al.*, 2006) were not detected in the Danube floodplain soils, indicating that there may be other ecological niches for their anamorphic stages.

The observed distribution of MOTUs in the vertical soil profile also suggests the existence of ecological factors that determine the proliferation of *Trichoderma*. *T. asperellum*, which was the dominant species in this study, was reproducibly associated with upper soil horizons, but its abundance did not correlate with the carbon and nitrogen content of the soil or pH of the soil solution. Also, the highest number of MOTUs for *T. asperellum* was found in the root-free A0 litter layer of the beech site, and we therefore suspect that the rhizosphere is not its prime habitat. However, it might be that the species is following other fungi that are highly abundant in soil litter and upper soil horizons. In contrast with that, *H*. *pachypallida* and *T. rossicum* were almost exclusively detected in the deepest soil horizons characterized by the sufficient organic carbon and the nearly complete absence of plant roots. Moreover, these species should be capable of growing well under conditions of nitrogen starvation as the amount of nitrogen in their habitat is one order of magnitude lower than that in the surface soil. Both the high species richness of the deepest mineral soil horizons (eight species in each sampling site) and their unique species compositions compared with upper soil layers suggest competitive relations between tribal relatives (and/or with other myco- and microbionts) rather than their associations with abiotic characteristics of these soil horizons.

The limited diversity of *Hypocrea/Trichoderma* in the studied soils may be attributed to the relatively high pH values (around 8) which could potentially prevent the development of other species. However, the same cosmopolitan and opportunistic species also dominated more acidic soils in Sardinia (pH~5; Migheli *et al.*, 2009) and were present in rhizospheric soil of *Coffea arabica* in Ethiopian highland forest (pH~5.5; Mulaw *et al.*, 2010), suggesting that this is not the controller of the diversity.

### *In situ* diversity confirms the sympatric speciation within *T. harzianum* *sensu* *lato* and related taxa

In previous studies that used cultivation-dependent methods to quantify *Hypocrea/Trichoderma* in various habitats, *T. harzianum* *sensu* *lato* represented the most dominant species (Druzhinina *et al.*, 2005; Migheli *et al.*, 2009; Zachow *et al.*, 2009; Druzhinina *et al.*, 2010b, for further references). In this study, we also detected a remarkable diversity of genetically sibling species from the *Harzianum*–*Catoptron* clade (Chaverri *et al.*, 2003; Druzhinina *et al.*, 2010b) in nearly all soil samples. Interestingly, in all mineral soil horizons of the beech forest site, *T. harzianum* *sensu stricto* coexisted with a member of *H*. ‘pseudoharzianum’ (Druzhinina *et al.*, 2010b), while in the aspen forest site, the latter was found together with other members of the *Harzianum*–*Catoptron* clade: *T. cerinum*, *T. pleuroticola* and *H. alni*. This finding suggests the sympatric speciation of *T. harzianum* *sensu stricto* and *H.* ‘pseudoharzianum’ (Druzhinina *et al.*, 2010b) which is also proposed for other pairs of sister species in the genus *Hypocrea/Trichoderma*, such as *H. jecorina* and *T. parareesei* (Druzhinina *et al.*, 2010a, Atanasova *et al.*, 2010) or *T. longibrachiatum* and *H. orientalis* (Druzhinina *et al.*, 2008). It is common to argue against the assignment of sympatric speciation modes to fungi as, although they may be distributed in overlapping ranges, they can occupy different microhabitats in those areas and therefore may still be spatially isolated (Burnett, 2003). Our findings show the non-random presence of MOTUs attributed to both *T. harzianum* *sensu stricto* and *H.* ‘pseudoharzianum’ in at least four soil habitats.

### *Hypocrea/Trichoderma* species express versatile effects on the presence of tribal relatives

In order to reconstruct the possible interactions between *Hypocrea/Trichoderma* species occurring in the same soil horizon, we selected a sample set of model strains (Table 4) and tested the effect of a fresh culture extract of one strain on the growth of the other in a pair-wise manner. *T. asperellum* and *H. atroviridis* were essentially inhibited by the presence of other taxa (up to 80 %). However, these species initially had faster growth rates of nearly twofold compared with others in the same experimental conditions (see Supplementary Data). Therefore, the inhibition put them in the growth rate range of all other species rather than stopping their development. These data suggest that when the fungus is able to accelerate its growth in the absence of direct competitors, it possesses a strong opportunistic potential to occupy a vacant ecological niche.

The true cases of growth inhibition were, nevertheless, detected when, for example, *H*. ‘pseudoharzianum’ grew very little on culture extracts of *H. atroviridis.* Several cases of strong stimulation of mycelial growth have been also observed. It is notable that the mycelial growth of *H.* ‘pseudoharzianum’ was stimulated by *T. asperellum* which is in agreement with the metagenomic data. In the BC horizon of aspen soil, these two species were codominant (18 and 23 MOTUs, respectively); the other abundant species in this horizon was *H. alni*, which is, in turn, stimulated in the presence of culture extracts of the two former species. Thus, our metagenomic data are largely supported by *in vitro* interaction experiments.

### *Hypocrea/Trichoderma* species may facilitate each other’s distribution

Our data also show a strong change in the pattern of conidiation in the presence of culture extracts from other species, which does not correlate with alterations in mycelial growth. Sporulation of *Trichoderma* is known to be triggered by light (Betina & Farkas, 1998). We have previously shown, however, that conidiation of *H. atroviridis* is strongly controlled by carbon metabolism while illumination plays only a secondary function in increasing the rate of conidia formation (Friedl *et al.*, 2008). As our experiments were done in darkness and the culture filtrates were all obtained from growth of the strains on the same carbon source, the fact that conidiation was stimulated by culture filtrates of closely related strains is very interesting. Nemcovic *et al.* (2008) reported that conidiation in *H. atroviridis* was accompanied by the increased production of eight-carbon compounds 1-octen-3-ol and its analogues 3-octanol and 3-octanone. When vapours of these compounds were applied individually to dark-grown colonies, even of other species, they elicited their conidiation at submicromolar concentrations. The authors concluded that the eight-carbon volatile organic compounds act as signalling molecules regulating development and mediating intercolony communication in *Hypocrea/Trichoderma*. In this study, we have not attempted to identify the particular stimulatory components; however, it is possible that some of the effects observed by us were due to such VOCs, but the multitude of bidirectional effects makes it rather unlikely that they are only due to a single component. In the present study we demonstrate that, for example, *T. rossicum*, which is most abundant in deep soil horizons ( = darkness), is not able to produce conidia under control conditions, while it starts to sporulate in the presence of almost all other cultures. We therefore conclude that many *Hypocrea/Trichoderma* species inhabiting the same microecological niche not only compete with one another but are also able to act synergistically, accelerating the sensing of abiotic factors and thus facilitating each other’s distribution. Such an assumption is in agreement with our metagenomic data.

### Biocontrol formulations may benefit from synergistic action of highly opportunistic *Hypocrea/Trichoderma* spp.

A community of highly opportunistic *Hypocrea/Trichoderma* has been detected in previous cultivation-dependent studies. Migheli *et al.* (2009) showed that among 16 species isolated from highly disturbed non-rhizosphere soils in Sardinia (Italy) *H.* ‘pseudoharzianum, *T. spirale*, *T. gamsii*, *T. hamatum* or *H. koningiopsis* consistently co-occurred. A similar result was shown in the pioneering metagenomic study on *Trichoderma* in agricultural soils when representatives of the *Harzianum*–*Catoptron* and *Hamatum* clades, which cover species known for their antagonistic potential, were recovered from the same samples (Hagn *et al.*, 2007). Zachow *et al.* (2009) applied metagenomic methods and traditional cultivation techniques to characterize the diversity of fungi in the rhizosphere of endemic plant species of Tenerife (Canary Islands) and showed the co-existence of extraordinarily highly antagonistic strains of *Hypocrea/Trichoderma*.

From a practical point of view, this demonstrates that the knowledge about infrageneric communities and interactions will be important for screening *Hypocrea/Trichoderma* strains to be used for the biological control of soil-borne plant-pathogenic fungi. Several strains showing synergism with one another may be combined in certain biocontrol formulations, while on the other hand, the indigenous *Hypocrea/Trichoderma* should not have antagonistic properties against the introduced biocontrol strain(s).

In this study, we investigated one of the last remaining undisturbed ecosystems in Central Europe. However, the diversity found in the national park largely resembles the *Hypocrea/Trichoderma* species composition in disturbed and agricultural soils (Migheli *et al.*, 2009; Hagn *et al.*, 2007). This finding indicates that the local highly opportunistic species (from those listed above) should be among the major taxa screened for the best *Trichoderma* biocontrol strains.

### Both ITS rRNA and *tef1* phylogenetic markers have limited applicability for *in situ* diversity studies using the high-throughput methods

Hagn *et al.* (2007) designed *Hypocrea/Trichoderma-*specific primers for the ITS1 fragment of the rRNA gene cluster. However, later studies showed that ITS1 is not sufficiently diagnostic as many species share the same allele. Meincke *et al.* (2010) set up genus-specific primers with the reverse primer located in a still polymorphic and indel-rich area of ITS2 30 bp upstream of the last genus-specific hallmark, which makes several species undetectable. The six Trirev primers presented in this study amplify the entire diagnostic region of ITS1 and 2 of all members of the genus. We have demonstrated the high specificity and selectivity of these primers as no MOTUs belonging to other fungi were recovered, but the two novel alleles of ITS1 and 2 were detected.

Hoyos-Carvajal *et al.* (2009) asserted the presence of paralogous copies of ITS1 and 2 in some *Hypocrea/Trichoderma* species. In order to test this, we constructed a clone library for randomly selected strains of *T. asperellum* (*n* = 30) and found only one allele (data not shown) of ITS1 and 2, confirming the absence of different alleles within a single genome.

The applicability of ITS1 and 2 for larger metagenomic studies using high-throughput sequencing methods remains questionable. On one hand, ITS-based quantification of species abundance in environmental samples will depend on the number of ITS copies in the respective genome, which may vary between different species. Furthermore, a growing number of *Hypocrea/Trichoderma* species share the same ITS allele making these taxa indistinguishable by this locus (Samuels *et al.*, 2006a, b; Jaklitsch *et al.*, 2006b; Druzhinina *et al.*, 2008; Atanasova *et al.*, 2010). Another disadvantage of ITS1 and 2 comes from the fact that these sequences are not appropriate for the design of species-specific qPCR primers, as most of their diagnostic zones are surrounded by long mononucleotide stretches and/or have an intolerable GC content (L. Bodrossy & I. S. Druzhinina, unpublished data). Gazis *et al.* (2011) compared the three communities of endophytic fungi and revealed that ITS alone usually underestimates the number of taxa predicted by other loci. Sequences for the highly polymorphic fourth large intron of the *tef1* gene are also available for the majority of *Hypocrea/Trichoderma* species, making it a good alternative to ITS1 and 2. This gene has a single copy in the genome and therefore becomes appropriate for quantitative assessments. However, the current public databases of Hypocreales *tef1* sequences do not yet allow the design of genus-specific primers. In this study, we attempted to develop species-specific *tef1* primers for those taxa that we either detected among MOTUs or could expect to be present in temperate soils (based, for example, on Jaklitsch, 2009, 2011). *In vitro* tests showed that only two out of 40 *in silico*-designed oligonucleotides are selective for target taxa, while others demonstrated unspecific affinity to the DNA of non-target species, probably due to the polymorphic secondary structures of the fragment. These results suggest that the *tef1* intron alone is also not appropriate for a large-scale metagenomic analysis of the genus due to its hypervariablity. The applicability of more conserved markers such as *rpb2* or *chi18-5* is currently being tested in our laboratory.
